# Supplementary material for: P-TRAP: a Panicle Trait Phenotyping tool
Source: BMC Plant Biol. 2013 Aug 29;13:122. doi: 10.1186/1471-2229-13-122 (PMC3848748; doi:10.1186/1471-2229-13-122)
Supplement: Additional file 7 — P-TRAP source code. The Netbeans project that contains the source code of the application. [file 1471-2229-13-122-S7.zip › javadoc.zip!/javadoc/org-biolag-particleprocessor/org/biolag/particleprocessor/Particle.CONTOUR_TYPE.html]

Particle.CONTOUR\_TYPE (ParticleProcessor)


JavaScript is disabled on your browser.


- Package
- Class
- Tree
- Deprecated
- Index
- Help

*org.biolag.particleprocessor 1.0*

- Prev Class
- Next Class

- Frames
- No Frames

- All Classes

- Summary:
- Nested |
- Enum Constants |
- Field |
- Method

- Detail:
- Enum Constants |
- Field |
- Method


org.biolag.particleprocessor

## Enum Particle.CONTOUR\_TYPE

- java.lang.Object
- - java.lang.Enum<Particle.CONTOUR\_TYPE>
  - - org.biolag.particleprocessor.Particle.CONTOUR\_TYPE

- All Implemented Interfaces:
  :   java.io.Serializable, java.lang.Comparable<Particle.CONTOUR\_TYPE>

  Enclosing class:
  :   Particle

  ---

    

  ```
  public static enum Particle.CONTOUR_TYPE
  extends java.lang.Enum<Particle.CONTOUR_TYPE>
  ```

- - ### Enum Constant Summary

    Enum Constants

    Enum Constant and Description

    `CONCAVE`

    `CONVEX`
  - ### Method Summary

    Methods

    | Modifier and Type | Method and Description |
    | --- | --- |
    | `static Particle.CONTOUR_TYPE` | `valueOf(java.lang.String name)` Returns the enum constant of this type with the specified name. |
    | `static Particle.CONTOUR_TYPE[]` | `values()` Returns an array containing the constants of this enum type, in the order they are declared. |

    - ### Methods inherited from class java.lang.Enum

      `clone, compareTo, equals, finalize, getDeclaringClass, hashCode, name, ordinal, toString, valueOf`
    - ### Methods inherited from class java.lang.Object

      `getClass, notify, notifyAll, wait, wait, wait`

- - ### Enum Constant Detail


    - #### CONVEX

      ```
      public static final Particle.CONTOUR_TYPE CONVEX
      ```


    - #### CONCAVE

      ```
      public static final Particle.CONTOUR_TYPE CONCAVE
      ```
  - ### Method Detail


    - #### values

      ```
      public static Particle.CONTOUR_TYPE[] values()
      ```

      Returns an array containing the constants of this enum type, in
      the order they are declared. This method may be used to iterate
      over the constants as follows:

      ```
      for (Particle.CONTOUR_TYPE c : Particle.CONTOUR_TYPE.values())
          System.out.println(c);
      ```

      Returns:
      :   an array containing the constants of this enum type, in
          the order they are declared


    - #### valueOf

      ```
      public static Particle.CONTOUR_TYPE valueOf(java.lang.String name)
      ```

      Returns the enum constant of this type with the specified name.
      The string must match *exactly* an identifier used to declare an
      enum constant in this type. (Extraneous whitespace characters are
      not permitted.)

      Parameters:
      :   `name` - the name of the enum constant to be returned.

      Returns:
      :   the enum constant with the specified name

      Throws:
      :   `java.lang.IllegalArgumentException` - if this enum type has no constant
          with the specified name
      :   `java.lang.NullPointerException` - if the argument is null


- Package
- Class
- Tree
- Deprecated
- Index
- Help

*org.biolag.particleprocessor 1.0*

- Prev Class
- Next Class

- Frames
- No Frames

- All Classes

- Summary:
- Nested |
- Enum Constants |
- Field |
- Method

- Detail:
- Enum Constants |
- Field |
- Method
